# Relationship between left ventricular geometric pattern and systolic and diastolic function in treated Nigerian hypertensives

**Published:** 2010-02

**Authors:** Adeseye A Akintunde, Patience O Akinwusi, O George Opadijo, Oluranti B Familoni

**Affiliations:** Division of Cardiology, Lautech Teaching Hospital, Osogbo, Osun State, Nigeria; Cardiology Clinic, Department of Internal Medicine III, Eberhard Karls University, Tubingen, Germany; Division of Cardiology, Lautech Teaching Hospital, Osogbo, Osun State, Nigeria; Division of Cardiology, Lautech Teaching Hospital, Osogbo, Osun State, Nigeria; Division of Cardiology, Olabisi Onabanjo University Teaching Hospital, Sagamu, Ogun State, Nigeria.

**Keywords:** hypertension, left ventricular hypertrophy, geometric pattern, systolic and diastolic function, association

## Abstract

**Introduction:**

Despite a high worldwide prevalence of left ventricular hypertrophy among black patients, the association of a specific left ventricular geometric pattern with left ventricular dysfunction is rare. The aim of this study was to explore the possibility of such an association in Nigerian hypertensives.

**Methods:**

This was a retrospective study consisting of 188 treated hypertensives. Echocardiography was used to allocate the patients to the following four groups: normal geometric pattern, concentric remodelling, eccentric hypertrophy and concentric hypertrophy.

**Results:**

The mean age of the study population was 55.95 ± 10.71 years. There were 75 females (39.9%). Concentric hypertrophy occurred in 72 (38.3%) patients and concentric remodelling in 53 (28.2%). Only 30 (16%) had a normal left ventricular geometric pattern. Hypertensive subjects with eccentric hypertrophy had the lowest ejection fraction, fractional fibre shortening and left ventricular ejection time but these did not reach statistical significance. The mean left atrial dimension was highest in the subjects with eccentric hypertrophy.

**Conclusion:**

In this study population of treated Nigerian hypertensives, concentric remodelling and hypertrophy were the predominant left ventricular geometrical patterns.

## Summary

Left ventricular hypertrophy is an important determinant of adverse cardiovascular events in subjects with hypertension.^1^ Racial predilection and associated cardiovascular disorders have been described, especially among blacks and African-Americans.^1^,^2^ Left ventricular hypertrophy (LVH) is associated with increased prevalence of heart failure (both systolic and diastolic), aortic root dilation, arrhythmias, sudden cardiac death and cerebrovascular events.^3^-^6^ In the presence of systolic and/or diastolic dysfunction, many other cardiovascular events could occur, such as arrhythmias and sudden cardiac death.

Several authors have demonstrated an increased cardiovascular risk associated with increased left ventricular mass (LVM).^7^,^8^ The processes that ultimately lead to heart failure, among other things, initially alter the left ventricular geometric pattern in various ways. Left ventricular geometric pattern is therefore an important prognostic factor in the epidemiology of cardiovascular diseases.

Blacks have been noted to have an increased prevalence of left ventricular hypertrophy and increased associated cardiovascular risk. The association of geometric patterns with left ventricular systolic and diastolic function has not been well studied. The development of heart failure in hypertensives with LVH results from depressed left ventricular systolic function and/or diastolic dysfunction. The deleterious effect of left ventricular remodelling may be an important determinant of progression to overt heart failure.^9^

The aim of this study was to determine any possible association between left ventricular dysfunction and left ventricular geometrical patterns in this population of treated Nigerian hypertensives, using echocardiography.

## Methods

This was a retrospective study among adult hypertensive subjects (≥ 18 years) who had had complete echocardiographic examination as part of their work-up in a teaching hospital. Hypertension was diagnosed with standard protocols when blood pressure was ≥ 140/90 mmHg on at least two occassions.^10^ Only patients receiving antihypertensive therapy were included. The following information was obtained: gender, age at the time of the echocardiogram, weight, height, calculated body mass index (BMI), and concurrent treatment with antihypertensive medication. BMI was calculated as weight/height^2^ (kg/m^2^).

In all patients, an abdominal ultrasound and urinalysis were performed. Echocardiography was done in all patients to document the presence or absence of LVH and also to document the left ventricular geometrical pattern. These studies were performed as part of the initial evaluation of hypertension or as part of the ongoing care of known hypertensive patients.

Echocardiography was performed on all the subjects using a Suis Apogee machine and a 3.5-MHz probe. Two-dimensional colour Doppler and pulse-wave Doppler were carried out. Echocardiography was done according to the American Society of Echocardiography guidelines.^11^ Two-dimensional guided M-mode echocardiograms were used for the measurement of the left ventricular internal dimension, interventricular septal thickness and left ventricular posterior wall thickness during diastole, according to the American Society of Echocardiography guidelines.^11^

LVM was calculated from measurements of the left ventricle (LV) using the equation:

LVM (g) = 0.81 [1.04 (interventricular septal thickness + posterior wall thickness + LV end-diastolic internal dimension)^3^ – (LV end-diastolic internal dimension)^3^] + 0.6.^12^

LVM index (LVMI) was calculated as LVM/height (m).^2.7^ Correcting LVM for height^2.7^ minimises the effect of gender, race, age and obesity on the validity of various parameters for the diagnosis of left ventricular hypertrophy.^13^,^14^ One adult criterion for LVH is LVMI > 51 g/m^2.7^. As reported by de Simone *et al.*,^15^ adult patients with hypertension and LVMI > 51 g/m^2.7^ have been found to be at a fourfold greater risk of cardiovascular morbidity outcomes. LV geometry was determined after calculation of the relative wall thickness (RWT) using the formula (2 × posterior wall thickness)/LV end-diastolic internal dimension.16 RWT was considered abnormal if it was ≥ 0.45.^16^

Four left ventricular geometric patterns were described: normal geometry, concentric remodelling, eccentric hypertrophy and concentric hypertrophy. LV geometry was defined as concentric hypertrophy (elevated LVMI and RWT), concentric remodelling (normal LVMI and elevated RWT), eccentric hypertrophy (increased LVMI and normal RWT) and normal geometry (normal LVMI and RWT). LV ejection fraction was calculated using Teichholz’s formula.^17^

Statistical analysis was done using the Statistical Package for Social Sciences, SPSS 15.0 (Chicago Ill.) Quantitative data were summarised using means ± standard deviation (SD) while qualitative data were summarised using percentages and proportions. The Student’s *t*-test and chi-squared test were used as appropriate for intergroup comparisons. Values of *p* < 0.05 were taken as statistically significant.

## Results

Table 1 shows the distribution of geometric patterns in the males and females in the study group. Abnormal geometry was more likely to occur among the males. Only 12.4% of males and 21.3% of females had normal geometry. The commonest abnormal geometry was concentric hypertrophy, occurring in 44.24% of males and 29.3% of females. As shown in Table 2, those with abnormal geometry were more likely to be older and had a longer duration of hypertension than those with normal geometry. They were also more likely to have a higher systolic and diastolic blood pressure than those with normal geometry.

**Table 1:** Distribution Of LV Geometry Patterns In Both Genders

	*Male (n = 113) (%)*	*Female (n = 75) (%)*	p
CH	50 (44.2)	22 (29.3)	< 0.05*
CR	32 (28.3)	21 (28.0)	> 0.05
EH	17 (15.0)	16 (21.3)	> 0.05
N	14 (12.4)	16 (21.3)	> 0.05
TOTAL	113 (100)	75 (100)	< 0.05*

CR: concentric remodelling, CH: concentric hypertrophy, EH: eccentric hypertrophy, N: normal geometry. *Statistically significant.

**Table 2:** Clinical Characteristics Of LV Geometry Patterns

*Variable*	*CH*	*EH*	*N*	*CR*	*ANOVA*
Age	57.89 ± 9.7	54.5 ± 10.7	56.6 ± 11.0	51.5 ± 12.1	0.05
Duration	8.0 ± 8.43	5.53 ± 7.3	6.17 ± 7.22	4.35 ± 5.1	0.165
BSA	1.83 ± 0.18	1.79 ± 0.19	1.81 ± 0.16	1.87 ± 0.18	0.40
SBP	152.13 ± 24.1	147.6 ± 21.4	144.5 ± 27.9	134.9 ± 17.5	0.02*
DBP	89.8 ± 13.2	90.69 ± 10.8	88.2 ± 11.85	86.4 ± 7.6	0.361

CR: concentric remodelling, CH: concentric hypertrophy, EH: eccentric hypertrophy, N: normal geometry, BSA: body surface area (g/m^2^), SBP: systolic blood pressure (mmHg), DBP: diastolic blood pressure (mmHg). *Statistically significant.

Table 3 shows the echocardiographic parameters in the study population. The mean left atrial dimension was highest among those with eccentric hypertrophy. Ejection fraction (EF) and fractional shortening (FS) were lower among subjects with abnormal geometry compared with those with normal geometry, although it was not statistically significant. Mean iso-volumic relaxation time (IVRT) was highest among subjects with eccentric hypertrophy.

**Table 3:** Echocardiographic Parameters For The Left Ventricular Geometric Patterns

*Variable*	*CH*	*CR*	*EH*	*N*	*p*
FS	34.8 ± 9.07	33.0 ± 8.95	31.74±13.01	37.92 ± 12.04	0.106
AOD	31.46 ± 4.5	31.0 ± 4.8	30.93 ± 4.51	29.62 ± 5.3	0.478
LAD	36.4 ± 6.06	33.36 ± 5.79	37.42 ± 9.92	33.6 ± 4.9	0.001*
SV	76.5 ± 29.23	45.16 ± 20.29	99.3 ± 36.1	71.87 ± 34.9	0.000*
MERAT	1.24 ± 1.65	0.94 ± 0.29	1.12 ± 0.57	1.13 ± 1.11	0.508
DT	208.5 ± 54.15	200.16 ± 47.7	192.7 ± 65.4	202.71 ± 55.2	0.310
IVRT	107.27 ± 28.3	100.4 ± 30.94	163.7 ± 29.61	102.47 ± 28.5	0.287
AVV_max_	1.21 ± 0.31	1.19 ± 0.39	1.17 ± 0.25	1.18 ± 0.31	0.737
AVVTI	26.8 ± 25.2	20.89 ± 5.87	19.84 ± 4.39	23.06 ± 16.3	0.188
AVPG_max_	6.26 ± 3.12	7.5 ± 11.67	6.4 ± 5.07	6.48 ± 6.9	0.71
AVV_mean_	0.78 ± 0.19	0.73 ± 0.18	0.73 ± 0.15	0.75 ± 0.17	0.371
LVET Dop	281.92 ± 46.5	274.37 ± 34.83	256.62 ± 61.3	293.12 ± 25.8	0.027*
LVPEP	97.25 ± 46.7	87.24 ± 29.5	102.7 ± 33.9	88.9 ± 20.6	0.234
LVSTI	0.34 ± 0.12	0.33 ± 0.12	0.40 ± 0.15	1.4 ± 0.46	0.140
LVDID	4.77 ± 0.66	3.83 ± 0.59	5.5 ± 0.94	4.7 ± 0.44	0.000*
LVISD	3.20 ± 0.83	2.7 ± 0.72	3.81 ± 1.48	2.95 ± 0.46	0.000*
IVSD	1.39 ± 0.23	1.27 ± 0.22	1.27 ± 0.21	1.07 ± 0.21	0.000*
PWTD	1.34 ± 0.18	1.20 ± 0.23	1.03 ± 0.16	0.87 ± 0.14	0.000*
LVM	269.25 ± 74.4	164.8 ± 38.6	264.94 ± 75.9	160.84 ± 36.0	0.000*
RWT	0.57 ± 0.09	0.65 ± 0.23	0.38 ± 0.09	0.38 ± 0.05	0.000*
LVMI	67.98 ± 17.5	41.44 ± 8.0	67.2 ± 19.17	38.23 ± 7.61	0.000*

CR: concentric remodelling, CH: concentric hypertrophy, EH: eccentric hypertrophy, N: normal geometry, BSA: body surface area (g/m^2^), SBP: systolic blood pressure (mmHg), DBP: diastolic blood pressure (mmHg), EF: ejection fraction (%), FS: fractional shortening (%), AOD: aortic root dimension (mm), LAD: left atrial dimension (mm), SV: stroke volume (ml), LVET (2D): left ventricular ejection time in 2-D echo (sec), MERAT: mitral e/a ratio, DT: deceleration time (sec), PHT: pressure at half time (mmHg), IVRT: isovolumic relaxation time (seconds), AVV_max_: maximum aortic valve pressure (mmHg), AVVTI: velocity time interval of aortic valve (mmHg), AVPG_max_: maximum aortic valve pressure gradient (mmHg), AVV_mean_: mean aortic valve pressure, LVET Dop: left ventricular ejection time with Doppler (sec), LVPEP: left ventricular pre-ejection pressure time (sec), LVSTI: left ventricular stroke–time interval gradient, LVIDD: left ventricular internal dimension in diastole (cm), LVISD: left ventricular internal dimension in systole (cm), IVSd: interventricular dimension in diastole (cm), PWTD: posterior wall thickness in diastole (cm), LVM: left ventricular mass (g), RWT: relative wall thickness, LVMI: left ventricular mass index (g/m^2.7^). *Statistically significant.

The mean left ventricular ejection time was reduced among those with abnormal geometry compared with those with normal geometry. Left ventricular dimensions (in both diastole and systole), left ventricular mass (LVM), relative wall thickness (RWT) and left ventricular mass index (LVMI) were statistically different between subjects with normal and abnormal geometry, as shown in Table 3. As shown in Table 4, hypertensive subjects with abnormal geometry had reduced left ventricular systolic function, as evidenced by reduced aortic valve and left ventricular output velocity–time intervals.

**Table 4:** Comparing Clinical And Echocardiographic Parameters Of Subjects With Normal Geometry With Those With Abnormal Geometry

*Variables*	*Normal geometry*	*Abnormal geometry*	*p*
Age	51.48 ± 12.1	56.53 ± 10.3	0.05
Duration	4.4 ± 5.13	6.79 ± 7.9	0.273
BSA	1.87 ± 0.18	1.82 ± 0.18	0.127
SBP	134.92 ± 17.45	149.23 ± 24.01	0.009*
DBP	86.4 ± 7.6	89.62 ± 12.0	0.417
EF	71.15 ± 13.97	67.88 ± 15.7	0.342
FS	37.92 ± 12.04	33.75 ± 10.03	0.057
AOD	29.62 ± 5.27	31.15 ± 4.63	0.298
LAD	33.55 ± 4.90	36.16 ± 7.3	0.070
SV	77.97 ± 36.2	71.55 ± 34.90	0.242
LVET	303.27 ± 38.94	273.4 ± 65.52	0.265
MERAT	1.31 ± 0.80	1.12 ± 1.19	0.49
DT	197.2 ± 55.96	203.44 ± 54.6	0.859
IVRT	94.46 ± 22.97	104.73 ± 29.73	0.081
AVV_max_	1.1 ± 0.19	1.19 ± 0.33	0.45
AVVTI	21.44 ± 4.92	22.24 ± 1.01	0.02*
AVPG_max_	5.16 ± 2.22	6.74 ± 7.6	0.55
AVV_mean_	0.70 ± 0.14	0.76 ± 0.18	0.461
LVET Dop	293.12 ± 25.81	274.95 ± 47.59	0.158
LVPEP	88.94 ± 2.06	95.36 ± 4.04	0.360
LVSTI	1.40 ± 4.67	0.34 ± 0.13	0.032*
LVIDD	4.66 ± 0.44	4.62 ± 0.95	0.774
LVISD	2.95 ± 3.16	3.16 ± 1.07	0.597
IVSD	1.07 ± 0.21	1.33 ± 0.24	0.000*
LVM	160.84 ± 35.96	234.48 ± 81.84	0.000*
RWT	0.38 ± 0.05	0.56 ± 0.18	0.000*
LVMI	38.24 ± 7.61	59.16 ± 20.03	0.000*

CR: concentric remodelling, CH: concentric hypertrophy, EH: eccentric hypertrophy, N: normal geometry, BSA: body surface area (m^2^), SBP: systolic blood pressure (mmHg), DBP: diastolic blood pressure (mmHg), EF: ejection fraction (%), FS: fractional shortening (%), AOD: aortic root dimension (cm), LAD: left atrial dimension (cm), SV: stroke volume (ml), LVET (2D): left ventricular ejection time in 2-D echo (sec), MERAT: mitral e/a ratio, DT: deceleration time (seconds), PHT: pressure at half time (mmHg), IVRT: isovolumic relaxation time (sec), AVV_max_: maximun aortic valve pressure (mmHg), AVVTI: velocity time interval of aortic valve (mmHg), AVPG_max_: maximum aortic valve pressure gradient (mmHg), AVV_mean_: mean aortic valve pressure, LVET Dop: left ventricular ejection time with Doppler (seconds), LVPEP: left ventricular pre-ejection pressure time (sec), LVSTI: left ventricular stroke–time interval gradient, LVIDD: left ventricular internal dimension in diastole (cm), LVISD: left ventricular internal dimension in systole (cm), IVSd: interventricular dimension in diastole (cm), PWTD: posterior wall thickness in diastole (cm), LVM: left ventricular mass (g), RWT: relative wall thickness, LVMI: left ventricular mass index (g/m^2.7^). *Statistically significant.

## Discussion

LVH has been recognised as an important predictor of adverse cardiovascular events, such as malignant arrhythmias, sudden cardiac death, heart failure and coronary heart disease.^18^-^20^ Abnormal left ventricular geometry has been shown recently to represent a subtle form of advanced LVH and is associated with systolic and diastolic dysfunction.^21^,^22^ These studies evaluated the prognostic significance of left ventricular geometrical patterns on the cardiovascular risk of hypertensive subjects. Concentric remodelling and concentric hypertrophy were reported in the Ochner studies to be associated with increased adverse cardiovascular risks.^21^ All-cause mortality has been reported to increase two-fold in concentric remodelling (similar to eccentric hypertrophy) and is further increased in concentric hypertrophy.^22^-^24^

This study demonstrated increased prevalence of left ventricular hypertrophy and abnormal left ventricular geometric patterns among treated Nigerian hypertensive subjects. This relatively increased prevalence has been documented among blacks worlwide. 25,26 Concentric remodelling and concentric hypertrophy were the commonest left ventricular geometric abnormalities in this study. This was similar to the findings from the Atherosclerosis Risk in Community (ARIC) study as reported by Fox *et al.*,^27^ who demonstrated that 65% of their hypertensive cohort had either concentric hypertrophy or concentric remodelling. Several studies have shown that the increased prevalence of LVH among blacks may be due to genetic susceptibility during their development. 28-31 The association of hypertension with left ventricular hypertrophy therefore calls for more aggressive treatment to reverse the adverse cardiovascular risk associated with it.

Subjects with eccentric hypertrophy had the lowest ejection fraction in this study. Others have reported similar associations among hypertensive subjects.^27^ The haemodynamic changes associated with eccentric hypertrophy caused increased left ventricular diastolic and systolic dimensions (as shown in Table 4) due to associated volume overload. This dilation of the left ventricle is an important risk factor for subsequent progressive reduction in left ventricular ejection fraction and heart failure. Those with eccentric hypertrophy also had the lowest left ventricular ejection time in this study. This was possibly due to ventricular chamber dilatation and consequently increased enddiastolic volume. Hence, the left ventricular output decreases and ultimately and progressively may lead to the development of heart failure.

The aortic valve velocity–time interval is an echocardiographic index of left ventricular output. It was lowest among those in this study with eccentric hypertrophy. Diastolic dysfunction including LV relaxation abnormality, pseudonormalisation (normal pulse wave of mitral valve inflow but with blunted or reversed pulmonary venous flow indicating increased left atrial pressure and restrictive filling) occurred in various LV geometric patterns. They have been associated with an additive effect on cardiovascular and all-cause mortality.

In this study, left atrial dimension was highest among subjects with eccentric hypertrophy. This pattern is also associated with other indices of diastolic dysfunction such as abnormal IVRT and deceleration time. Left atrial dimension has been shown to be a good index of left ventricular diastolic dysfunction.^32^

Although, the mean e/a ratio and deceleration time across the groups were not statistically different, the differing left atrial dimension is a good index of the presence of diastolic dysfunction among the subjects. These findings suggest that abnormal LV geometry (especially eccentric hypertrophy) was associated with systolic and diastolic dysfunction among treated hypertensive Nigerians. In regional left ventricular function studies such as tissue Doppler studies, cardiac MRI may demonstrate more significant evidence of left ventricular dysfunction.

It is important to note that LVH prevalence is still high, as revealed among treated hypertensive subjects. Similar studies from Ibadan, Nigeria also revealed a high prevalence of LVH and abnormal geometry among treated hypertensives. However, the study reported a higher prevalence among women.^33^ This was despite the use of antihypertensive drugs, although drug adherence and compliance were not assessed in this study. Only 28% of newly diagnosed hypertensive subjects were shown to have normal left ventricular geometry in a study from the same centre.^34^ The management of LVH and abnormal geometry therefore seems to be an important therapeutic goal to prevent the progression of the condition.

## Conclusion

This study further highlights the relatively increased prevalence of left ventricular hypertrophy and abnormal left ventricular geometric pattern among treated hypertensive Nigerian subjects. Hypertensive subjects with eccentric hypertrophy had reduced ejection fraction, fractional shortening and left ventricular ejection time than those with other geometric patterns. They also had increased left atrial dimension. Eccentric and concentric hypertrophy were the commonest forms of left ventricular geometry among the subjects.
